# Molecular characterization, gene expression and functional analysis of goldfish (*Carassius auratus* L.) macrophage colony stimulating factor 2

**DOI:** 10.3389/fimmu.2023.1235370

**Published:** 2023-08-01

**Authors:** Moussa Gouife, Ziqi Ban, Xinyuan Yue, Jianhu Jiang, Jiasong Xie

**Affiliations:** ^1^ School of Marine Sciences, Ningbo University, Ningbo, Zhejiang, China; ^2^ Agriculture Ministry Key Laboratory of Healthy Freshwater Aquaculture, Zhejiang Institule of Freshwater Fisheries, Huzhou, Zhejiang, China; ^3^ Key Laboratory of Aquacultural Biotechnology, Ministry of Education, Ningbo University, Ningbo, China; ^4^ National Engineering Research Laboratory of Marine Biotechnology and Engineering, Ningbo University, Ningbo, Zhejiang, China

**Keywords:** fish, macrophage colony-stimulating factor 2, progenitors, hematopoiesis, cytokine

## Abstract

**Background:**

Macrophage colony-stimulating factor 2 (*MCSF-2*) is an important cytokine that controls how cells of the monocyte/macrophage lineage proliferate, differentiate, and survive in vertebrates. Two isoforms of *MCSF* have been identified in fish, each exhibiting distinct gene organization and expression patterns. In this study, we investigated a goldfish *MCSF-2* gene in terms of its immunomodulatory and functional properties.

**Methods:**

In this study, goldfish were acclimated for 3 weeks and sedated with TMS prior to handling. Two groups of fish were used for infection experiments, and tissues from healthy goldfish were collected for RNA isolation. cDNA synthesis was performed, and primers were designed based on transcriptome database sequences. Analysis of gfMCSF-2 sequences, including nucleotide and amino acid analysis, molecular mass prediction, and signal peptide prediction, was conducted. Real-time quantitative PCR (qPCR) was used to analyze gene expression levels, while goldfish head kidney leukocytes (HKLs) were isolated using standard protocols. The expression of gfMCSF-2 in activated HKLs was investigated, and recombinant goldfish *MCSF-2* was expressed and purified. Western blot analysis, cell proliferation assays, and flow cytometric analysis of HKLs were performed. Gene expression analysis of transcription factors and pro-inflammatory cytokines in goldfish head kidney leukocytes exposed to rgMCSF-2 was conducted. Statistical analysis using one-way ANOVA and Dunnett’s *post hoc* test was applied.

**Results:**

We performed a comparative analysis of *MCSF-1* and *MCSF-2* at the protein and nucleotide levels using the Needleman-Wunsch algorithm. The results revealed significant differences between the two sequences, supporting the notion that they represent distinct genes rather than isoforms of the same gene. Sequence alignment demonstrated high sequence identity with *MCSF-2* homologs from fish species, particularly *C. carpio*, which was supported by phylogenetic analysis. Expression analysis in various goldfish tissues demonstrated differential expression levels, with the spleen exhibiting the highest expression. In goldfish head kidney leukocytes, gfMCSF-2 expression was modulated by chemical stimuli and bacterial infection, with upregulation observed in response to lipopolysaccharide (LPS) and live *Aeromonas hydrophila*. Recombinant gfMCSF-2 (rgMCSF-2) was successfully expressed and purified, showing the ability to stimulate cell proliferation in HKLs. Flow cytometric analysis revealed that rgMCSF-2 induced differentiation of sorted leukocytes at a specific concentration. Moreover, rgMCSF-2 treatment upregulated *TNFα* and *IL-1β* mRNA levels and influenced the expression of transcription factors, such as *MafB*, *GATA2*, and *cMyb*, in a time-dependent manner.

**Conclusion:**

Collectively, by elucidating the effects of rgMCSF-2 on cell proliferation, differentiation, and the modulation of pro-inflammatory cytokines and transcription factors, our findings provided a comprehensive understanding of the potential mechanisms underlying gfMCSF-2-mediated immune regulation. These results contribute to the fundamental knowledge of *MCSF-2* in teleosts and establish a foundation for further investigations on the role of gfMCSF-2 in fish immune responses.

## 1 Introduction

*MCSF*, also named colony stimulating factor 1 (*CSF-1*), is a growth factor that has multiple effects on cells such as monocytes, macrophages, and bone marrow progenitors (1, 2). Indeed, *MCSF* plays an essential role in controlling the maturation, shape, survival, and function of tissue macrophages and osteoclasts (3). All of *MCSF*’s effects are triggered by one high-affinity transmembrane receptor (*MCSF-R*), which is generated by the c-fms protooncogene (4). Through binding to its receptor, *MCSF* upregulates the expression of additional markers of macrophage differentiation and promotes the expansion of macrophage progenitor populations (5). By aiding in its chemotactic, phagocytic, and destructive activities, *MCSF* supports monocyte and macrophage defense mechanisms (6). Therefore, it is clear that *MCSF* plays a crucial role in regulation of downstream immunological responses, early defense against invading pathogens, tissue homeostasis, and tissue repair based on its effects on this myeloid lineage (3).

In mammals, there are three different biologically active *MCSF* isoforms, a heavily glycosylated form that associates with the extracellular matrix, a membrane-bound form that can be released by the action of proteases, and a soluble form (7–9). Endothelial cells are responsible for producing the soluble form of *MCSF* that circulates in the blood, while other forms of *MCSF* are produced locally in the tissues (10, 11). Moreover, many cell lines produce *MCSF in vitro*, either naturally or after being induced. *In vivo*, *MCSF* has been hypothesized to enhance M2 polarization because of homeostatic expression and the overall M2-like phenotype of resident macrophage populations under normal conditions (12, 13). Several diseases, including inflammatory and autoimmune conditions, as well as viral and bacterial infections, have been linked to *MCSF* in mammals (14–16).

Macrophage-like cells may be found in almost all multicellular creatures and their roles have been substantially preserved throughout evolution. While *MCSF*s have been extensively studied and documented in mammals, this is not the case for lower vertebrates (17). Goldfish (*Carassius auratus*) was the first non-mammalian species to have their *MCSF* gene identified and characterized (18). Subsequent discoveries included grouper (*Epinephelus coioides*) (19, 20), zebrafish (*Danio rerio*), rainbow trout (*Oncorhynchus mykiss*) (21, 22), Japanese flounder (*Paralichthys olivaceus*) (17). Although numerous studies have been conducted on *MCSF*s (19, 21, 23–25), only a few have pointed to a role for teleost *MCSF*s in fish immune systems (20, 25). Furthermore, limited functional studies have been conducted, in particular the effects of *MCSF* on monocyte differentiation and proliferation in goldfish (26). In addition, recombinant trout *MCSF* may boost CXCR3 expression in head kidney macrophages and promote proliferation of kidney head leukocytes (21). Recent studies in Japanese flounder (*Paralichthys olivaceus*) have looked at the immunomodulatory and physiologic effects of *MCSF* (17). Despite the fact that Wang and co-authors found two *MCSF* genes in fish with distinct gene organization and expression patterns (21), it is surprising that previous studies have focused more on *MCSF-1* compared to *MCSF-2*.

Currently, studies concerning the functional characterization of *MCSF-2* in teleosts are limited. Therefore, the present study will, examine the immunomodulatory ability and functional properties of *MCSF-2* in teleosts, and specifically in the goldfish.

## 2 Materials and methods

### 2.1 Fish

Goldfish (*Carassius auratus* L.) of the Crucian carp variety were obtained from a local aquatic market in Ningbo and housed in the aquatic facilities of the Laboratory of Aquatic Animal Diseases and Immunology, University of Ningbo. Fish were acclimated for a minimum of 3 weeks, fed daily with pellets, and maintained at room temperature under a simulated natural photoperiod using a circulating water system. Randomly selected male or female goldfish, approximately 2 years old, measuring between 10 and 15 cm in length, were used for the experiments. Before handling, the fish were sedated with a solution of TMS (tricaine methane sulphonate) at 40 to 50 mg/L in water. The experimental protocol, identified as Protocol # NBU20210046, was approved by the Ningbo University Council of Animal Care, and all studies were conducted in accordance with the established regulations.

### 2.2 Bacterial infection

To prepare the *A. hydrophila* strain for infection experiments, TSA plates were used to incubate during 24 h at 28°C, the bacterial culture. The culture was then transferred to TSB medium and shaken for 12 h at 28°C. The sediment was rinsed three times with phosphate-buffered saline (PBS) after centrifugation at 1,000 *g* for 5 minutes at 4°C. Two groups of fish were used for the infection assay: a control group and an experimental group. The control group received 100 µL of sterile PBS, while the experimental group received 100 µL of sterile PBS containing *A. hydrophila* at a concentration of 1 × 10^8^ CFU/mL.

### 2.3 RNA isolation and cDNA synthesis

Tissues including spleen (Sp), kidney (Ki), brain (Br), heart (He), liver (Li), muscle (Mu), intestine (In) and gills (Gi) were taken from 6 healthy goldfish. Following homogenization, tissues were extracted using Trizol reagent (Omega Biotech, China) to isolate RNA. After being checked for purity and concentration, RNA samples were stored at -80°C and analyzed using a Nanodrop 2000 spectrophotometer (Thermo Fisher Scientific, USA). A Prime-Script RT Reagent Kit with gDNA Eraser (Takara, Japan) was used to reverse transcribe RNA and make first-strand cDNA. Until needed, cDNA samples were kept at -20°C.

### 2.4 Gene cloning, and sequence analysis

The primers were designed according to the sequences obtained from the previously generated transcriptome database (27). All primers employed in cloning and expression of gfMCSF-2 are provided in **Supplementary Table 1** (**Table S1**). For cDNA template preparation, we extracted RNA from goldfish kidney, followed by utilizing the PerfectStart Green qPCR SuperMix (TransGen, China). To amplify the product, we followed the thermocycling parameters consisting of a first step of denaturation at 95°C for 2 min, followed by 30 rounds of amplification at 95°C for 30 s, 55 for 45 s, 72°C for 2 min for 30 s, a last step of extension at 72°C for 12 min. Sequence study of gfMCSF-2 nucleotides and predicted amino acids, we used the NCBI BLAST program and the ExPASy server Molecular Biology (http://us.expasy.org). The Compute *p*I/Mw tool at ExPASy (http://www.expasy.ch/) was used to predict the molecular mass and isoelectric point of the putative gfMCSF-2, and SignalP was utilized to predict the signal peptide. The alignment of several sequences was performed by CLUSTAL-W (http://www.genome.jp/tools-bin/clustalw), and images were generated using ESPript 3.0 (http://espript.ibcp.fr/ESPript/cgi-bin/ESPript.cgi). The p-distance method of the MEGA 11 program, with values expressed as percentages, was employed to perform the gfMCSF-2 phylogenetic analysis, 10,000 bootstrap replicates.

### 2.5 Real-time quantitative PCR analysis

Goldfish specific *MCSF-2* primers were created with Primer Express software (Applied Biosystems, USA). Tissues and cell groups from six different goldfish (*n* = 6) were analyzed using the ABI QuantStudio 5 instrument (Thermo Fisher Scientific, USA) for Q-PCR. Thermocycling settings were 95°C for 10 min, 40 cycles of 15 s at 95°C, and 60°C for 1 min. GraphPad Prism (GraphPad Software, USA) was utilized for statistical analysis and graphing purposes. Each gene’s transcription levels were quantified using a standard curve and standardized to *EF-1α*. Expression variations were computed by comparing the experimental group’s mean expression level to the control groups.

### 2.6 Isolation of goldfish head kidney leukocytes

HKLs were isolated from goldfish using standard protocols (28). Briefly, the kidneys were excised and kept in ice-cold NMGFL-15 medium. The tissues were then homogenized, and the cell solution was suspended on top of 51% Percoll using a 400 g centrifuge at 4°C for 25 min to remove debris. At the 51% Percoll/medium junction, the cells were collected and washed twice with incomplete medium before resuspending the HKLs in complete medium. Viable HKLs were counted using the trypan blue exclusion method before use in experiments.

### 2.7 Analysis of *MCSF-2* expression in activated goldfish HKLs

To prepare heat-killed and alive bacteria, the methods previously described were used (29). Six goldfish were used for this study (*n* = 6), and leukocytes from their kidneys were utilized to investigate gfMCSF-2 expression. Leukocytes were cultured at a density of 2 x 10^6^ cells/mL for a volume of 500 μL in each well on a 24 plate, then exposed to either 1 x 10^7^ CFU/mL of live *A. hydrophila*, 1 x 10^7^ CFU/mL of heat-killed *A. hydrophila*, or 10 µg/mL of LPS (Sigma L2630). A total of 1 x 10^6^ cells were used in 500 µL of complete NMGFL-15 media for each treatment group. After the cells were challenged, they were kept alive at 26 °C for 6 and 12 h. The TranScript Uni All-in-One First-Strand cDNA synthesis Supermix (Transgen, China) was used to synthesize cDNA from various cell types’ RNA. The reference gene *EF-1α* was used to determine gfMCSF-2’s relative expression levels.

### 2.8 Prokaryotic expression and scale up production of recombinant goldfish recombinant *MCSF-2*

Goldfish *MCSF-2* ORF excluding singnal peptide was amplified by PCR using gene-specific primers that included 5’-end *BamH* I and *Hind* III insertions (**Table S1**). Restriction enzymes *BamH* I and *Hind* III were used to thoroughly digest the PCR result. (Thermo Fisher Scientific, USA), then ligated to the pET32a (+) vector digested with *BamH* I/*Hind* III. This resulted in the creation of the recombinant plasmid pET32a-gfMCSF-2, which were subsequently converted into competent *E. coli* (BL21/DE3) cells (TransGen, China). To express the RgMCSF-2 proteins, 0.1 mM IPTG induction was carried out at 16°C overnight, and recombinants proteins expression was analyzed by SDS-PAGE. Purification of the recombinant protein was accomplished by using Ni-NTA Sefinose Resin (Sangon, China) as directed by the manufacturer. As a control protein in subsequent experiments, the pET32a (+) vector containing a thioredoxin tag without an insert was expressed and purified in the same manner. Subsequently, a ProteoSpin Endotoxin removal column (Norgen Biotek, USA) was used to purify the recombinant protein after it was dialyzed overnight at 4°C in 1 x PBS. Micro BCA Protein Assay Kit (Beyotime, China) was used to determine protein content.

### 2.9 Purification of recombinant proteins

The recombinant proteins of the genes studied were transformed into *E. coli* Transetta (DE3) (TransGen, China). After plates were kept at 37°C for one night, cells were grown in LB+AMP broth until the OD_600_ hit 0.6–0.8. A final concentration of 0.1 mM IPTG was added to the cell culture, which was then kept at 37°C for 3–5 hours, pelleted, and kept at -80°C overnight or until use. The pellet was dissolved with ultra-sonication for 10 minutes after being mixed with Solution I (150 mM NaCl, 1 mM EDTA, 2 M Urea, Triton (1X) 0.5%, pH=7.9). After centrifugation, Ultra-sonication was repeated for 10 minutes after the addition of solution II (150 mM NaCl, 20 mM Tris-HCL, 30 mM Imidazole, 8 M Urea, Triton (1X) 0.2%, pH= 7.9). To acquire the supernatants, we centrifuged the samples at 7,500 rpm for 20 minutes at 4°C after the prior procedure. For protein separation, a Nickel-nitrilotriacetic acid (Ni-NTA) column (Sangon Biotech, China) was used. Solution II was used to clean the Ni-NTA column, and the sample was left in the column overnight at 4°C while it was constantly shaken. The sample was passed through the Ni-NTA column and washed the next day with solution III (NaCl 150 mM, Tris-HCL 20 mM, Imidazole 20 mM, Urea 6 M, Triton (1X) 0,1%) to remove any residual material. Proteins were recovered from the column after being eluted with Elution solution (10 mM NaCl, 2 mM Tris-HCL, 100 mM Imidazole, 1.5 M Urea).

### 2.10 Renaturation of purified recombinant proteins

Subsequently, the denatured proteins were renatured overnight in 10 times the volume of renaturation buffer (5 mM EDTA, 2 mM oxidized glutathione, 4 mM reduced glutathione, 50 mM sodium borate, pH = 8.5). To eliminate the excess urea and imidazole molecules, the renatured protein was added to a 20 kDa dialysis bag and stored overnight at 4°C in 1 X PBS. The following day, PEG20000 [HO(CH_2_CH_2_O)_n_H] was used to remove excess buffer, and protein was collected in new tubes. Western blot analysis confirmed the presence of protein, and the concentration was determined using a BCA Protein Assay Kit (Beyotime, China) and stored at -80°C for future use.

### 2.11 Western blot analysis

The expressed and purified recombinant rgMCSF-2 and recombinant thioredoxin (rTrx) were analyzed by western blot. Sodium dodecyl sulfate-polyacrylamide gel electrophoresis (SDS-PAGE) was used to separate the samples, and then they were transferred to a nitrocellulose membrane at 200 mA for 2 h. Primary antibody [anti-His (1:3,000)] was incubated with the membrane at 4°C overnight after being blocked with 5% skim milk in TBST buffer (0.05% Tween-20 in TBS, pH 7.4) for 2 hours at room temperature. The membrane was rinsed and incubated for 1 hour the next day, with a secondary antibody [HRP-conjugated goat anti-mouse IgG (1:5,000)] for 1 h at room temperature. Detection of the recombinant proteins was conducted by Tanon 5200 Chemiluminescent Imaging System (Tanon, China).

### 2.12 Detection of cell proliferation of HKLs with MTT assay

Goldfish HKLs suspension (1 x 10^5^ cells/mL) was plated in 96-well dishes and treated with rgMCSF-2 at various concentrations (0.1, 0.25, 0.5, 0.75 and 1 μg/mL) for 24 h and 48 h. As a control, rTrx was added at 5 μg/mL. After centrifugation at 230 g for 5 min, MTT was added to each well, and the plates were kept in the incubator for an extra 4 h. The formazon salts were dissolved by adding DMSO, and the optical density was measured at 540 nm using a microplate reader (Bio-rad, USA). Three independent experiments were conducted.

### 2.13 Flow cytometric analysis of HKLs

After being isolated, HKLs from goldfish were seeded at densities of 1×10^6^ cells/well on a 6-well plate. Cells were exposed to concentrations of rgMCSF-2 ranging from 0.5 to 1 μg/mL. As a control, we employed rTrx at a final concentration of 5 μg/mL. The samples were then analyzed using a Fluorescence-activated cell sorter (FACS) Calibur flow cytometer (Becton Dickinson, USA) 24 h and 48 h after treatment, while the plates were incubated at 20°C. Cellular forward (size) and side (internal complexity) scatter light patterns were measured across all treatment groups (*n* = 3).

### 2.14 Transcription factor and pro-inflammatory cytokine gene expression in goldfish leukocytes triggered by rgMCSF-2

To evaluate the effect of rgMCSF-2 on goldfish leukocytes, six different fish were used to create primary cultures that were then exposed to either 5 μg/mL of rgMCSF-2 or 5 μg/mL of rTrx (as a control). TRIzol reagent was used to extract RNA from the cells after they had been incubated at 26°C for 6 h and 12 h. Quantitative PCR was used to analyzed the mRNA levels of a variety of transcription factor genes and pro-inflammatory cytokine relative to the housekeeping gene *EF-1α*. In a total volume of 500 μL of complete NMGFL-15 media, 1 × 10^6^ cells were used for each treatment group.

### 2.15 Statistical analysis

All experimental data were analyzed using one-way ANOVA, and then compared to the control and treatment groups using Dunnett’s *post hoc* test *P* < 0.05 was used as the threshold for significance.

## 3 Results

### 3.1 Sequence analysis and characterization of goldfish *MCSF-2*

The goldfish *MCSF-2* nucleotide sequence has been deposited to the Genbank under the Accession No. OQ459355. The goldfish *MCSF-2* cDNA transcript exhibited a distinct ORF comprising 804 base pairs (bp) and encoding a protein of 267 aa. Notably, it possessed a signal peptide of aa (**Figure S1**). Comparative analysis of goldfish *MCSF-1* and *MCSF-2* coding region nucleotide sequences was performed using Clustal multiple sequence alignment (**Figure S2**). These findings highlight the differential characteristics of the gfMCSF isoforms, emphasizing the unique attributes of gfMCSF-2 in relation to its precursor gfMCSF-1. Additionally, sequence alignment of *MCSF-1* and *MCSF-2* protein sequences indicated obvious difference between the two proteins (**Figure S3**). These results strongly suggest that *MCSF-1* and *MCSF-2* represent distinct proteins with notable variations.

**Figure 1:**
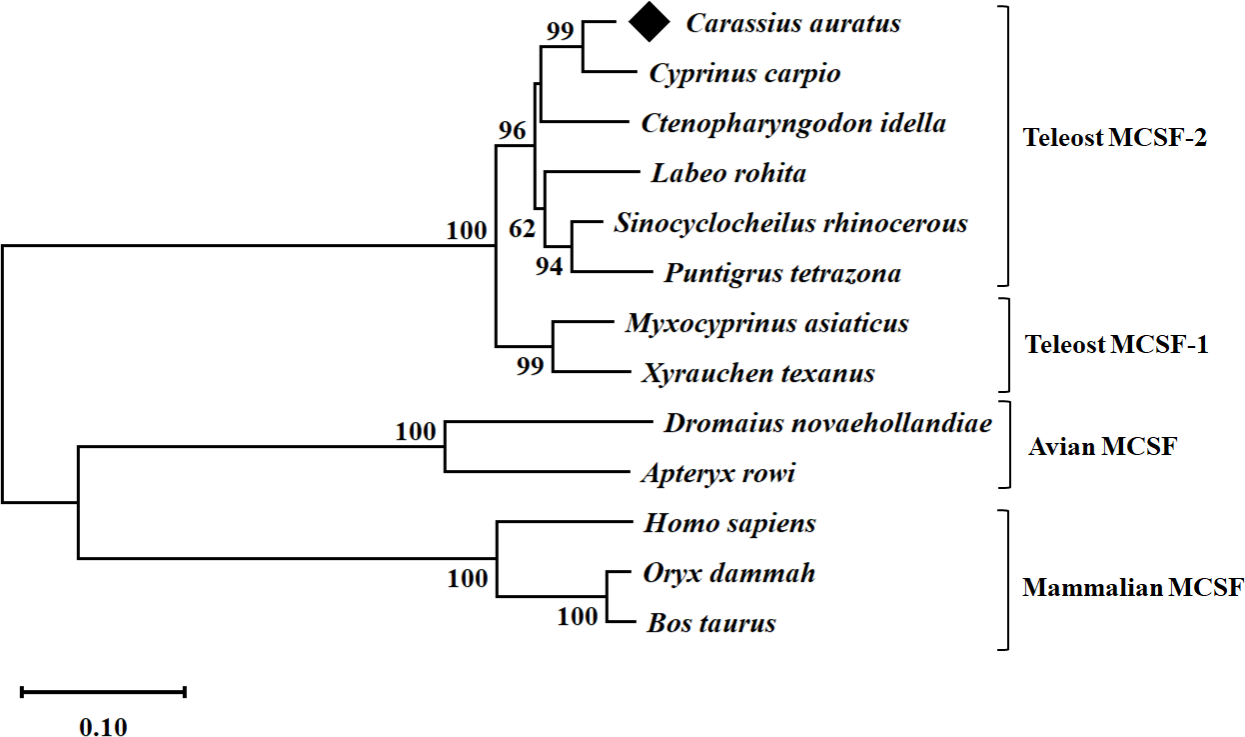
Phylogenetic analysis of goldfish *MCSF-2* from different species. The evolutionary history was inferred using the p-distance method. The optimal tree is shown. The percentage of replicate trees in which the associated taxa clustered together in the bootstrap test (10,000 replicates) are shown next to the branches. The tree is drawn to scale, with branch lengths in the same units as those of the evolutionary distances used to infer the phylogenetic tree. The evolutionary distances were computed using the p-distance method and are in the units of the number of base substitutions per site. All ambiguous positions were removed for each sequence pair (pairwise deletion option). Evolutionary analyses were conducted in MEGA 11.

A conservative CSF-1 superfamily domain was predicted and observed between positions 38 and 155 aa. The theoretical molecular weight (Mw) of full gfMCSF-2 protein was 30.57 kDa, and its isoelectronic point (*p*I) was 6.14. Based on multiple sequence alignment, gfMCSF-2 has the highest sequence identity with *MCSF-2* homologs from *C. carpio* (94%), *S. rhinocerous* (92%), *C. idella* (89%), and the lowest identity (18-20%) with those from mammals, and aves (**Table S2**). In addition, the secondary structure of gfMCSF-2 is similar to that of human *MCSF-2* (**Figure S4**). In order to investigate the phylogenetic relationships of gfMCSF-2, we retrieved homologous amino acid sequences from various teleost fishes through the NCBI database. These sequences were utilized to construct a phylogenetic tree encompassing multiple vertebrate species. The MEGA 11 software and the *p*-distance method were employed to generate a neighboring joining tree. The resulting phylogenetic analysis, depicted in **Figure 1**, demonstrated the closest evolutionary relationship between gfMCSF-2 and the *MCSF-2* of *C. carpio*. Additionally, when examining the phylogenetic analysis of goldfish *MCSF-1* and *MCSF-2* alongside other species, we observed a clear distinction between two distinct groups, as illustrated in **Figure S5**. This observation implies a genetic consistency between these species throughout their evolutionary history, as reflected in the phylogenetic tree.

### 3.2 *MCSF-2* expression in healthy goldfish tissues

**Figure 2:**
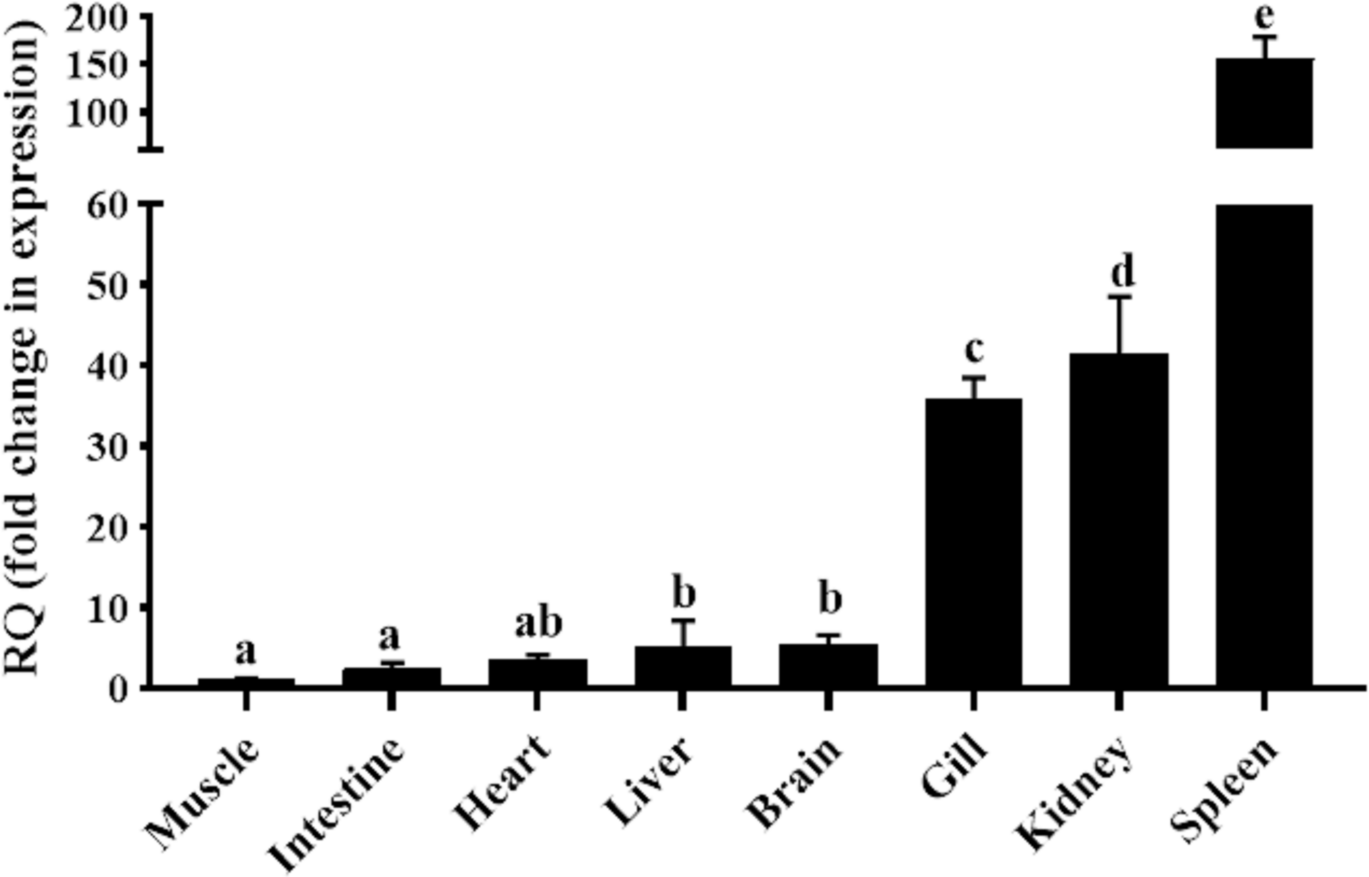
Expression analysis of *MCSF-2* in tissues obtained from normal goldfish. Analysis of the relative tissue expression was done using tissues from six fish (*n* = 6), qPCR was done in triplicate for each tissue. The expression of *MCSF-2* was relative to endogenous control gene, *EF-1α*. All results were normalized to lowest expression tissue (muscle). Statistical analysis was performed using one-way ANOVA followed by Dunnett’s *post-hoc* test. Different letters above each bar denote statistically different (*P* < 0.05), and the same letter indicates no statistical differences between groups.

qRT-PCR was carried out in eight different tissues of healthy goldfish, with the muscle tissue serving as the reference tissue for the analysis. As shown in **Figure 2**, the mRNA expression of gfMCSF-2 was found in all tested organs. The spleen had the highest mRNA level of MCSF-2, according to the analysis of gfMCSF-2 expression, followed by the kidney and the gills (**Figure 2**). The mRNA levels in the muscle, intestine, and heart were found to be relatively lower than other tissues (**Figure 2**).

### 3.3 Analysis of goldfish *MCSF-2*, *TNFα* and *IL-1β* expression in HKLs treated with chemical stimuli and bacteria

**Figure 3:**
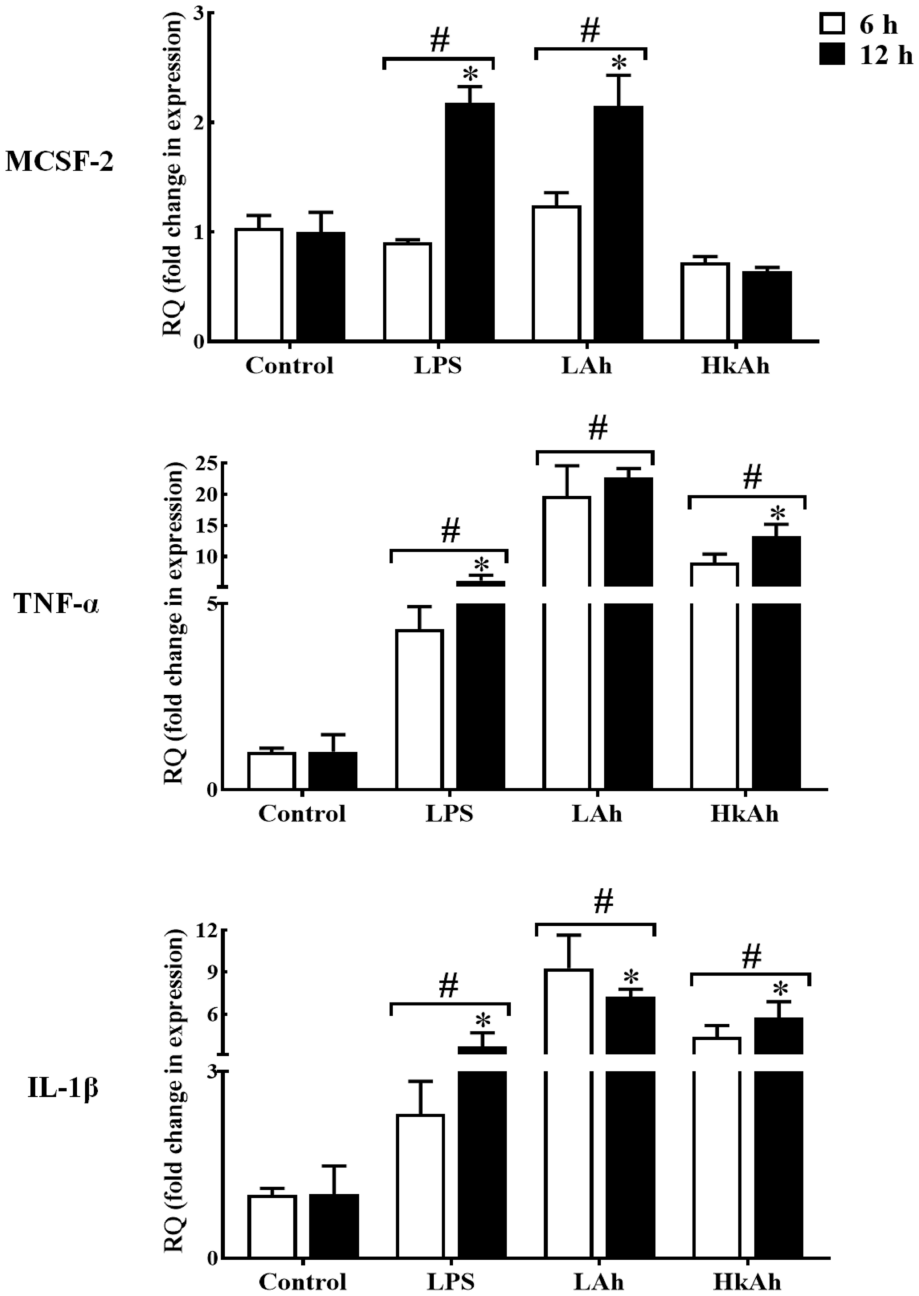
Quantitative expression of goldfish *MCSF-2*, *TNF-α* and *IL-1β* in leukocytes treated with different stimuli. The expression of *MCSF-2*, *TNF-α* and *IL-1β* in HKLs treated with either rTrx as a control, or lipopolysaccharide (LPS), heat-killed and live *A. hydrophila*. The expression of *MCSF-2*, *TNF-α* and *IL-1β* were examined relative to the endogenous control gene, elongation factor 1 alpha (*EF-1α*). The pound sign (#) indicates the significant difference at *P* < 0.05 compared to the control at 6 h or 12 h time point. The asterisk (*) denotes the significant difference at *P* < 0.05 between 6 h and 12 h treated groups.

As for *TNFα* and *IL-1β* which were used in this experiment as a positive control, a significant increase of these cytokines’ mRNAs level was observed after treatment of goldfish HKLs with the various stimuli at 6 h and 12 h when compared with the control groups (**Figure 3**).

### 3.4 Recombinant goldfish *MCSF-2* expression and purification

We expressed rgMCSF-2 in *E. coli* and purified it using the Ni-NTA Sefinose Resin protein purification system to investigate the functional activities of gfMCSF-2. A single band of approximately 50.27 kDa was observed in SDS-PAGE analysis of Commassie blue stained rgMCSF-2, which included the N-terminal six-histidine tag and Trx fusion protein (**Figure S6**). **Figure S6** shows that the recombinant protein was also verified by Western blotting using an anti-His antibody.

### 3.5 Effects of rgMCSF-2 treatment on cell proliferation of goldfish HKLs

**Figure 4:**
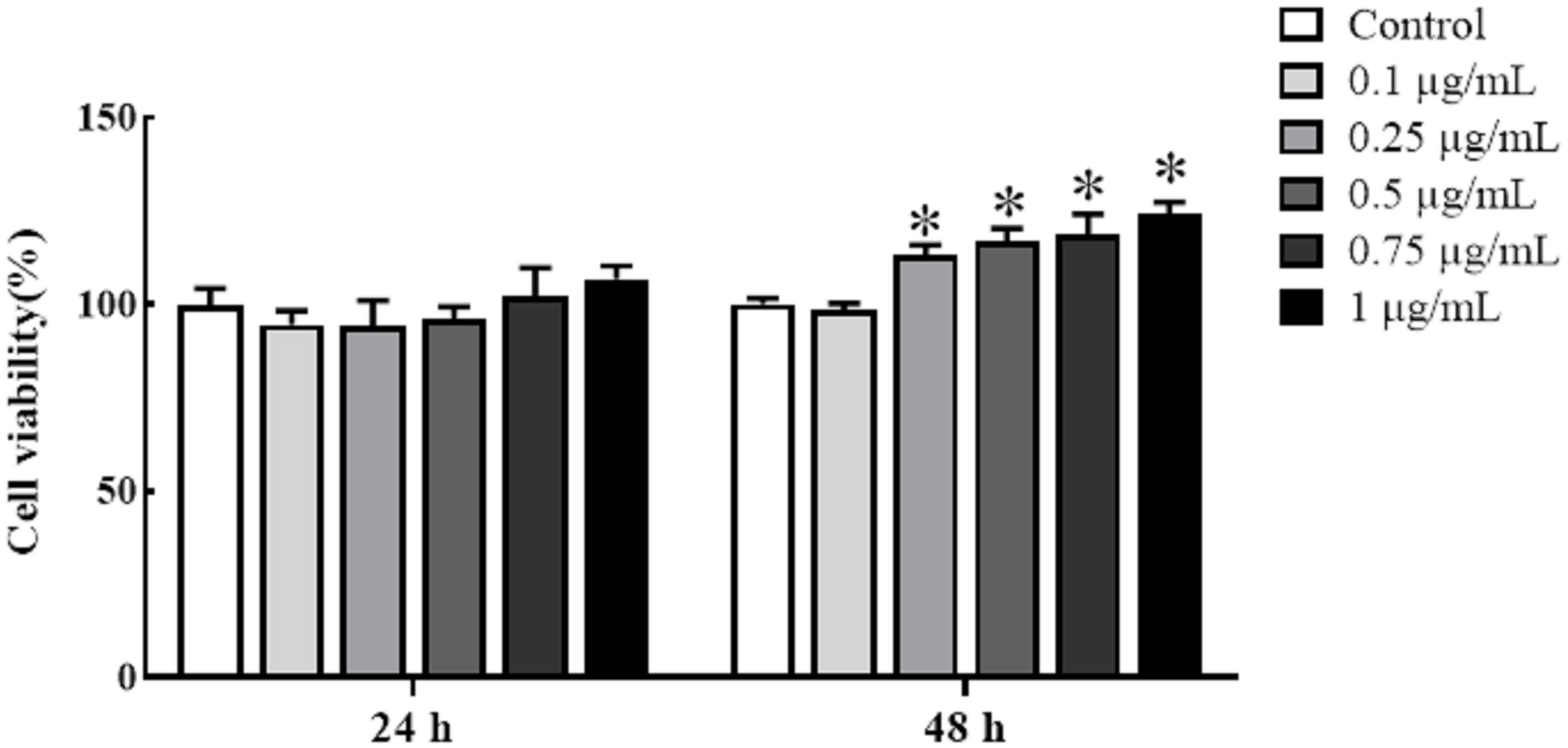
Effects of rgMCSF-2 on the proliferation of goldfish leucocytes using MTT assay. Goldfish head kidney leucocytes were treated with or without rgMCSF-2 at different concentrations for 24 h and 48 h. The proliferation of the cells was determined by MTT assay. Data are presented as means ± SEM (*n* = 3). Significance is denoted by (*) compared to the reference sample (*P* < 0.05).

Cultured HKLs were exposed to either rTrx as a control at a final concentration of 5 μg/mL or rgMCSF-2 at concentrations of 0.1, 0.25, 0.5, 0.75, and 1 μg/mL to determine whether rgMCSF-2 might stimulate the proliferation of immune cells. Treatment with rgMCSF-2 for 24 h resulted in mild cell proliferation at 0.75 and 1 μg/mL, while treatment for 48 h showed significant cell proliferation at all concentrations tested except 0.1 μg/mL (**Figure 4**).

### 3.6 Flow cytometric results of goldfish HKLs

**Figure 5:**
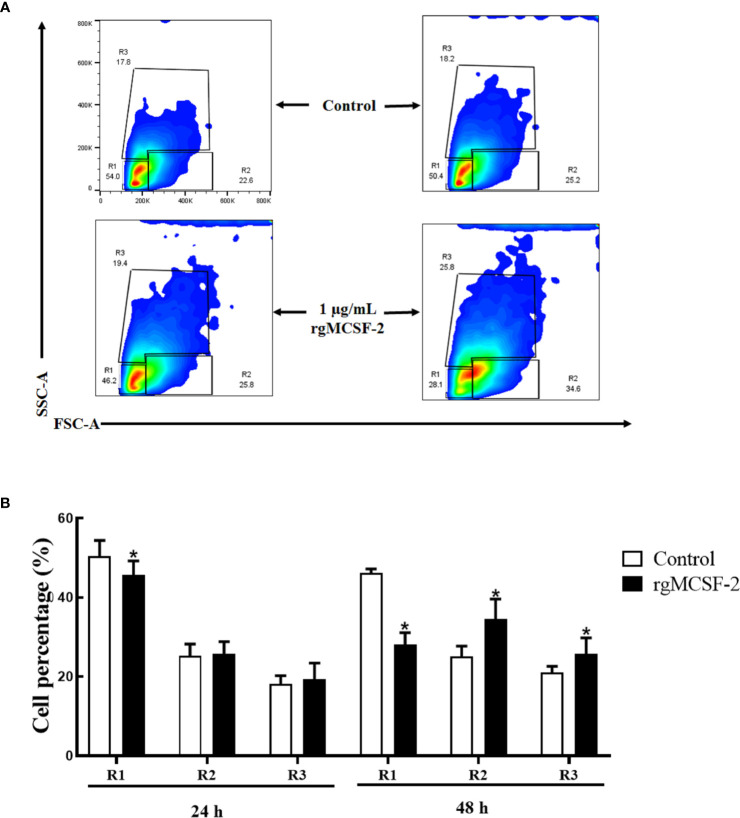
Flow cytometric analysis showing the differentiation of goldfish progenitors, after treatment with 1 µg/mL rgMCSF-2. **(A)** Analysis shows the results obtained at 24 h and 48 h taken from three individual fish. **(B)** Histogram shows the percentage of cells in R1, R2, and R3 after treatment with 1 µg/mL rTrx as a control and of rgMCSF-2 for 24 h and 48 h. Data are presented as means ± SEM (*n* = 3). Significance is denoted by (*) compared to the reference sample (*P* < 0.05).

Sorted goldfish leucocytes were treated with recombinant goldfish *MCSF-2* at concentrations of 0.1, 0.25, 0.5, 0.75, and 1 μg/mL. Over the course of two days, the influence of rgMCSF-2 on cell differentiation in isolated subpopulations of sorted leukocytes was evaluated by monitoring these cells with flow cytometry every 24 h. The differentiation of sorted goldfish leukocytes was unaffected after treatment at the concentrations of 0.1, 0.25, and 0.5 μg/mL of rgMCSF-2. Sorted leukocytes, however, were induced to differentiate when treated with rgMCSF-2 at a concentration of 1 μg/mL (**Figure 5**).

### 3.7 Proinflammatory cytokine analysis in rgMCSF-2 treated goldfish HKLs

**Figure 6:**
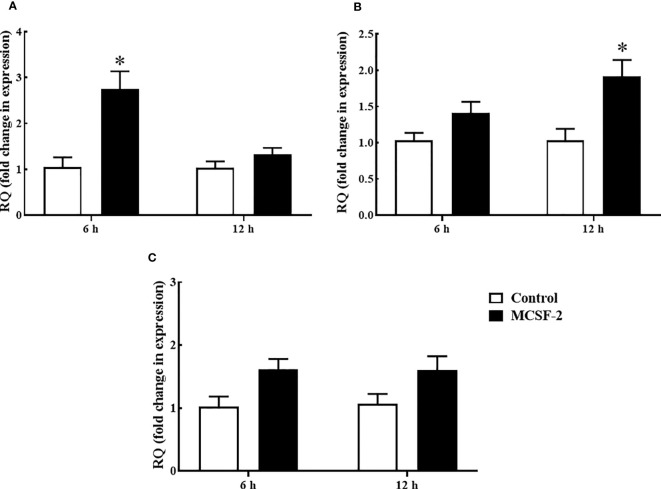
Quantitative expression analysis of goldfish proinflammatory cytokines *TNF-α* **(A)**, *IL-1β* **(B)** and *IFNγ* **(C)** in HKLs treated with 5 µg/mL rgMCSF-2 for 6 h and 12 h. The expression of goldfish *MCSF-2* was examined against the endogenous control gene, elongation factor 1 alpha (*EF-1α*). Expression values were normalized to those seen in rTrx -treated cells. Results are the mean ± S.E.M. of primary leukocyte cultures established from six individual fish (*n* = 6). The asterisk (*) denotes the significant difference at P < 0.05 between the control and MCSF2-treated groups.

Goldfish leukocyte cultures were treated with rgMCSF-2, and we measured the induction of *TNFα*, *IL-1β* and *IFNγ* protein levels and gene expression. The pro-inflammatory cytokines *TNFα* (**Figure 6A**) and *IL-1β* (**Figure 6B**) had their mRNA levels significantly upregulated after 6 h and 12 h, respectively, of treatment with 5 μg/mL rgMCSF-2 in goldfish HKLs. *IFNγ* mRNA production, however, was not significantly affected by rgMCSF-2 (**Figure 6C**).

### 3.8 Transcription factor analysis in rgMCSF-2 treated goldfish HKLs

**Figure 7:**
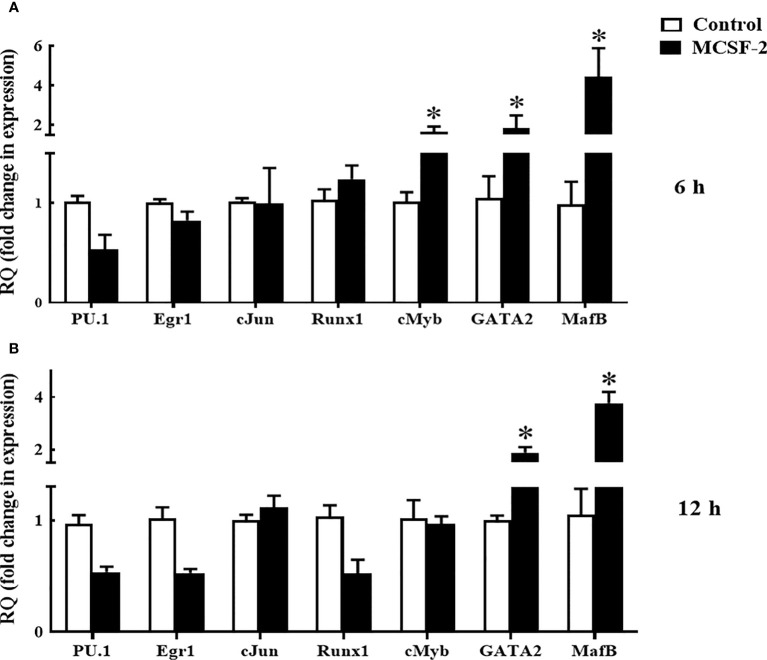
Quantitative expression analysis of goldfish transcriptions factors in HKLs treated with 5 µg/mL rgMCSF-2 for 6 h **(A)** and 12 h **(B)**. The expression of goldfish *MCSF-2* was examined against the endogenous control gene, elongation factor 1 alpha (*EF-1α*). Expression values were normalized to those seen in rTrx -treated cells. Results are the mean ± S.E.M. of primary leukocyte cultures established from six individual fish (*n* = 6). The asterisk (*) denotes the significant difference at P < 0.05 between the control and MCSF2-treated groups.

Next, we looked at whether or not rgMCSF-2 had a role in controlling transcriptional factors. After 6 h of treatment with 5 μg/mL rgMCSF-2, HKLs showed elevated mRNA levels of transcriptional regulators *MafB*, *GATA2*, and *cMyb* (**Figure 7A**). After 12 h of treatment, mRNA levels of transcriptional regulators *MafB* and *GATA2* were also dramatically increased in HKLs (**Figure 7B**). In contrast, there were no obvious changes in *cJun*, *Egr1*, *PU.1*, or *Runx1* mRNA levels over time (**Figure 7**).

## 4 Discussion

*MCSF* is a multifunctional cytokine that promotes the survival, proliferation, and differentiation of macrophages and their precursors, also it plays a significant role in the antimicrobial immune response in a wide variety of species, including mammals, birds, and teleosts (4, 30). Wang and coauthors found two *MCSF* genes in fish, both of which are differentially expressed and have distinct gene structures (21). Although *MCSF* has also been identified in teleosts, the majority of the research to date has concentrated on *MCSF-1*. This research described the expression and functional properties of the goldfish *MCSF-2* gene.

From our previously generated transcriptome database, the goldfish *MCSF-2* gene was cloned and isolated. The gfMCSF-2 cDNA transcripts contained an ORF of 804 bp encoding 267 aa, which is nearly identical to the *MCSF-2* genes of rainbow trout (276 aa) and zebrafish (284 aa) (21), indicating that the structural features of *MCSF-2* have been conserved in vertebrates throughout evolution. The observed differences in both protein and nucleotide sequences provide valuable insights into the evolutionary divergence and genetic variation of *MCSF-1* and *MCSF-2*. The substantial gaps in the alignment results suggest the presence of insertions or deletions, indicating structural differences between the two proteins. These variations may contribute to distinct functional properties or regulatory mechanisms associated with gfMCSF-2. The phylogenetic tree clearly demonstrated that gfMCSF-2 is a member of the teleost *MCSF-2* family, showing a high level of cluster with the *MCSF-2* proteins from other fishes. Following the same pattern as previous research (17, 21), gfMCSF-2 was clustered with fish from the class Actinopterygii, and more specifically with *Cyprinus carpio*, indicating that *MCSF-2* in teleosts has been evolutionarily conserved.

Gills, kidneys, and spleen had relatively higher gfMCSF-2 expression, while the heart, intestine, and muscle had the lower mRNA level. These findings matched those of previous studies on *MCSF-2* tissue distribution in *O. mykiss* and *D. rerio*, which found that *MCSF-2* expression was typically highest in the kidney and spleen (21). Therefore, kidney is where most bony fishes’ hematopoiesis and monocyte/macrophage formation takes place, the spleen also being a particularly macrophage-rich organ (31).

To investigate *MCSF-2* activators in fish, we first treated HKLs isolated from goldfish primary kidneys with LPS and fish pathogen *A. hydrophila* for 6 h and 12 h. Our results showed that the gfMCSF-2 mRNA level was increased in the LPS and live *A. hydrophila* groups compared to the control group after 12 h. Also, the results of the LPS and live *A. hydrophila* treatments between 6 and 12 h were significantly different. However, gfMCSF-2 mRNA expression was reduced after treatment with heat-killed *A. hydrophila*, although this change was not statistically significant at 6 h and 12 h. These findings are in line with previous studies in higher vertebrates that have demonstrated the stimulatory effect of LPS on *MCSF* production. For example, in human monocytes, LPS stimulation leads to increased production of *MCSF*. Similarly, in murine macrophages, LPS treatment induces the secretion of *MCSF* (5). This highlights the role of LPS as a potent inducer of *MCSF* production in higher vertebrates. In mammals, LPS has been shown to induce the expression of *MCSF* in various cell types (32). In an experimental rabbit model, Eichinger and colleagues found that *MCSF* was significantly up-regulated following valvular surgery and *Staphylococcus aureus* infection (33). Mice exposed to *Leishmania donovani* amastigote antigens have their serum *MCSF* production stimulated (34). Furthermore, the induction of *MCSF* by LPS is associated with the recruitment and activation of macrophages, which play a crucial role in the innate immune response. *MCSF* serves as a chemoattractant for monocytes and macrophages, promoting their differentiation and activation (11). This recruitment and activation of macrophages are essential for the clearance of pathogens and the initiation of an appropriate immune response. Unfortunately, there is no relative research reports on *MCSF-2* regarding immune defense against pathogenic infection in teleost. Therefore, our results show that HKLs of goldfish treated with live *A. hydrophila* and LPS significantly increased the expression of gfMCSF-2, implying that *MCSF-2* may be involved in fish resistance to bacterial and viral infections, as well as in mammalian defense.

Mammalian *MCSF* was discovered to be a hematopoietic growth factor that promotes the growth, survival and differentiation of monocytes, macrophages, and their progenitors in bone (35–37). In teleost, goldfish *MCSF-1* elicited functional responses including increased macrophage differentiation, proliferation from hematopoietic phagocytosis, chemotaxis, precursors, and production of antimicrobial reactive oxygen and nitrogen intermediates (18, 23, 26). Administration of *MCSF-1* to goldfish *in vivo* increased the quantity of bloodstream-circulating monocytes (26). However, the role of *MCSF-2* in teleost immune-related cells has not yet been fully reported. Hence, our study investigated the potential effects of rgMCSF-2 on immune cell proliferation and differentiation in goldfish. We found that treatment with rgMCSF-2 resulted in both cell proliferation and differentiation. Our study investigated the potential effects of rgMCSF-2 on immune cell proliferation and differentiation in goldfish. We found that treatment with rgMCSF-2 resulted in both cell proliferation and differentiation. In terms of cell proliferation, our results demonstrated a time-dependent effect of rgMCSF-2 treatment. After 24 h of treatment, mild cell proliferation was observed at concentrations of 0.75 and 1 μg/mL, while significant cell proliferation was evident at all concentrations tested except 0.1 μg/mL after 48 h. These findings suggest that rgMCSF-2 has the capacity to stimulate immune cell proliferation in goldfish. Similar stimulatory effects of *MCSF* on cell proliferation have been reported in higher vertebrates. In mammals, *MCSF* has been shown to promote the proliferation of mononuclear phagocytes, including macrophages and osteoclasts (11). For instance, in murine macrophages, *MCSF* treatment induces their proliferation and enhances their survival (13, 37). These studies support the notion that the proliferative effects of *MCSF-2* on immune cells are conserved across vertebrates. Regarding cell differentiation, our results indicated that treatment with rgMCSF-2 at concentrations of 0.1, 0.25, and 0.5 μg/mL did not significantly affect the differentiation of sorted goldfish leukocytes. However, at a concentration of 1 μg/mL, rgMCSF-2 induced the differentiation of sorted leukocytes. This observation suggests that rgMCSF-2 can drive the differentiation of specific immune cell subpopulations in goldfish. The capacity of *MCSF* to induce cell differentiation has been well-documented in higher vertebrates. In mammals, *MCSF* is a critical factor for the differentiation of monocytes into macrophages and their subsequent activation (11). Moreover, *MCSF* is involved in osteoclastogenesis, promoting the differentiation of precursor cells into functional osteoclasts (13, 37). These findings indicate the conserved role of *MCSF-2* in regulating immune cell differentiation and function across different vertebrate species.

Furthermore, it has been discovered that *MCSF-1* upregulates goldfish antimicrobial responses by inducing proinflammatory gene expression (23). However, there are no relevant research reports regarding the role of *MCSF-2* in regulating pro-inflammatory responses in teleosts. To explore the function of rgMCSF-2 in the stimulation of pro-inflammatory cytokine production, rgMCSF-2 was given to goldfish HKLs, and mRNA and protein levels of *IFNγ, IL-1β*, and *TNFα* were measured. After treating goldfish HKLs with 5 μg/mL of rgMCSF-2, the pro-inflammatory cytokines *TNFα* and *IL-1β* mRNA levels increased significantly. These findings mirrored those from studies in mammals, which found that *MCSF* can trigger the production of various cytokines, including *TNFα* (38), and those from studies on teleost, which found that *MCSF-1* can stimulate the expression of pro-inflammatory genes including *TNFα* and *IL-1β* in goldfish and Japanese flounder (17, 23). Consequently, these findings point to the possibility that *MCSF-2* in fish is essential for pro-inflammatory responses by activating *TNFα* and *IL-1β*.

In order to gain a better understanding of hematopoietic cells in teleosts and goldfish in particular, we looked at the effect rgMCSF-2 has on the differentiation of progenitor cell populations by measuring the mRNA levels of relevant transcription factors. Thus, myeloid transcription factor *MafB, GATA2*, and *cMyb* mRNA levels were significantly upregulated in HKLs after treatment with rgMCSF-2, as is typical for cell lines derived from monocytes and macrophages (39). *MafB* has been reported to stimulate the differentiation of myeloblasts into monocytes and macrophages (40–43) and to be highly expressed in monocytes/macrophages (29). Despite the dearth of research on the function of *MafB* in teleost myelopoiesis, the goldfish model revealed the presence of a *MafB* transcript and demonstrated that *MafB* mRNA increased with macrophage development in the goldfish primary renal macrophage culture system (44). Activation of myeloid genes, as well as the maintenance and expansion of multipotent progenitor cells, are all processes in which *GATA2* plays a role as a transcription factor (39). An evolutionarily conserved function for *cMyb* in hematopoiesis is shown by the fact that it is a marker of definitive HSCs in embryonic zebrafish (45) and is required for the formation of all blood cells (46). These findings further support our prior hypothesis that rgMCSF-2 plays an important role in myeloid lineage cell proliferation and differentiation.

In conclusion, we provided insights into the functionality of gfMCSF-2 by demonstrating that rgMCSF-2 not only promoted the proliferation and differentiation of primary head kidney cells but also increased the expression of proinflammatory cytokines such as *TNFα* and *IL-1β* in HKLs. Significant increases in the mRNA levels of the transcriptional regulators *MafB, GATA2*, and *cMyb* were also triggered by rgMCSF-2. Overall, our results indicated that gfMCSF-2 may have a significant function in goldfish progenitor cell growth, differentiation, and immunological response. Thus, our findings have laid the groundwork for the functional characterization of *MCSF-2* in teleosts.

## Data availability statement

The datasets presented in this study can be found in online repositories. The names of the repository/repositories and accession number(s) can be found in the article/**Supplementary Material**.

## Ethics statement

All fish experiments were conducted in accordance with the guidelines set by Council of Animal Care of Ningbo University under the Protocol # NBU20210046.

## Author contributions

MG, ZB designed the research, completed most experiemnts and wrote the manuscript. MG, XY analyzed the data and provided further input. JJ and JX generated the research idea and revised the manuscript. All authors contributed to the article and approved the submitted version.
