# Supplementary material for: Molecular characterization, gene expression and functional analysis of goldfish (Carassius auratus L.) macrophage colony stimulating factor 2
Source: Front Immunol. 2023 Aug 1;14:1235370. doi: 10.3389/fimmu.2023.1235370 (PMC10431942; doi:10.3389/fimmu.2023.1235370)
Supplement: Supplementary file 1 [file DataSheet_1.docx]

***Supplementary Material***

**Molecular characterization, gene expression and functional analysis of goldfish (*Carassius auratus*** **L.) macrophage colony stimulating factor 2**

**Moussa Gouife, Ziqi Ban, Xinyuan Yue, Jianhu Jiang, Jiasong Xie ^*^**

*** Correspondence:** Jiasong Xie: xiejiasong@nbu.edu.cn

**Supplementary Table 1.** The primers used in the study

| **Names** | **Sequence 5’-3’** | **Application** |
| --- | --- | --- |
| **T7 Promoter** | TAATACGACTCACTATAGGG | Vector specific primers |
| **T7 Terminator** | GCTAGTTATTGCTCAGCGG | Vector specific primers |
| **Vali-MCSF-2** | F- TGTGGAGCATTTCTTATCCTACTCA | RT-PCR primers |
|  | R- TGACGCTGCAGCTTCTTTCTC |  |
| **Q-MCSF-2** | F- GTCCTTGCAAACATGCCATCACTA | Quantitative PCR primers  for goldfish genes |
|  | R- AACACACCACGCTCAGGTGTTTT |  |
| **pET32A--MCSF-2** | F-GCGGATCCATGGACATCCCGGGTCCTTGCAAAC | Prokayotic expression primers |
|  | R-GCAAGCTTTCAGTTAGAAGGCAGTGGAGAGGTGTG |  |
| **IFNγ** | F- GAAACCCTATGGGCGATCAA | Quantitative PCR primers of proinflammatory cytokines |
|  | R- GTAGACACGCTTCAGCTCAAACA |  |
| **IL-1β1** | F- GCGCTGCTCAACTTCATCTTG |  |
|  | R- GTGACACATTAAGCGGCTTCAC |  |
| **TNFα1** | F- CATTCCTACGGATGGCATTTACTT |  |
|  | R- CCTCAGGAATGTCAGTCTTGCAT |  |
| **TNFα2** | F- TCATTCCTTACGACGGCATTT |  |
|  | R- CAGTCACGTCAGCCTTGCAG |  |
| **cMyb** | F- GGGCTTACGGATGCATTAAAGA | Quantitative PCR primers  for transcription factors |
|  | R- GAGCAGGGATGCCTTCCA |  |
| **Egr1** | F- TATCCTAACCGGCCAAGCAAGACA |  |
|  | R- TCTGCCGCATGTGGATCTTAGTGT |  |
| **MafB** | F- CCAACATCAACACCAACAATACG |  |
|  | R- GACCCGGGCGAGATAGGA |  |
| **GATA2** | F- CACCATCCCATCCCAACCTA |  |
|  | R- GCTTTTGCATTTGGGTGTGA |  |
| **PU.1** | F- TCGCCTCCTGTTGTTGATGTAA |  |
|  | R- CAGTCGCAGTCCTCCGTTAGA |  |
| **Runx1** | F- TCAAGGTAGTTGCCCTTGGTGATA |  |
|  | R- TTGAGGAGGGTTTGTGAAGACGGT |  |
| **cJun** | F- CGGCGATCCGGTTCCT |  |
|  | R- CCTCTCTCCCCCATCGACAT |  |
| **CEBPα** | F- ATCAAACAAGAGCCTCGGGAGGAA |  |
|  | R- TGGATTTCCCTCGATCGCCAATCT |  |

**Supplementary Table 2.** The MCSF-2 protein sequences used for multiple alignments and phylogenetic analysis.

| **Abbreviations** | **Species** | **Identity (%)** | **Accession no.** |
| --- | --- | --- | --- |
| **Hom** | *Homo sapiens* | 20 | XP_054190518.1 |
| **Dro** | *Dromaius novaehollandiae* | 18 | XP_025967824.1 |
| **Apt** | *Apteryx rowi* | 20 | XP_025912480.1 |
| **Ory** | *Oryx dammah* | 20 | XP_040088035.1 |
| **Bos** | *Bos taurus* | 19 | XP_005204128.1 |
| **Lab** | *Labeo rohita* | 88 | XP_050974121.1 |
| **Pun** | *Puntigrus tetrazona* | 88 | XP_043102636.1 |
| **Sin** | *Sinocyclocheilus rhinocerous* | 92 | XP_016417149.1 |
| **Cte** | *Ctenopharyngodon idella* | 89 | AGV15504.1 |
| **Cyp** | *Cyprinus carpio* | 94 | XP_042618571.1 |
| **Myx** | *Myxocyprinus asiaticus* | 84 | XP_051508809.1 |
| **Xyr** | *Xyrauchen texanus* | 77 | XP_051949609.1 |
| **Car** | *Carassius auratus* | - | OQ459355 |

**Supplementary Figure 1.** Nucleotide and predicted sequence of goldfish *MCSF-2* cDNA. The open reading frame (ORF) start and stop codons are shown in red with an asterisk in the termination codon. The predicted signal peptide is shown in yellow. A conserved domain of the CSF-1 superfamily is shown in green. The conserved cysteine residue has been underlined.


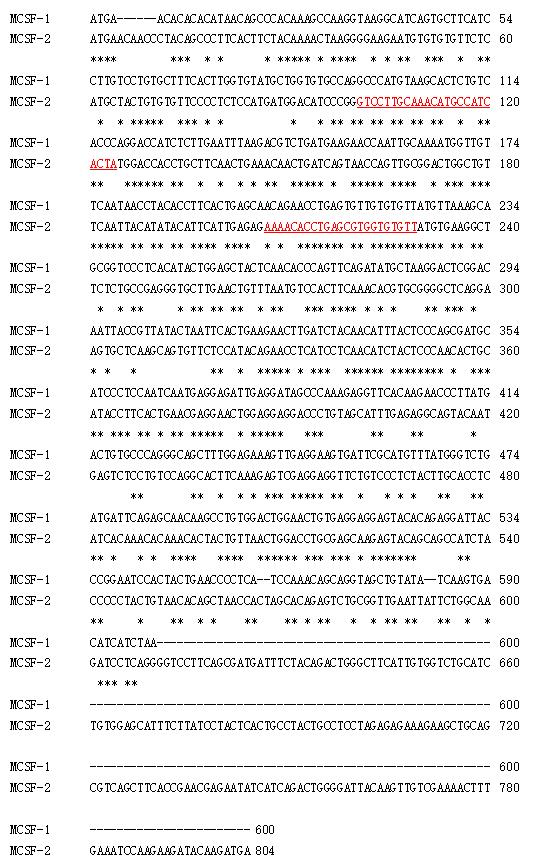


**Supplementary Figure 2.** Comparative analysis of goldfish *MCSF-1* and *MCSF-2* coding region nucleotide sequences is performed using Clustal multiple sequence alignment. The forward and reverse qPCR primers used in this study are underlined in red. The asterisk (*) indicates the same nucleotide sequence between two sequences.


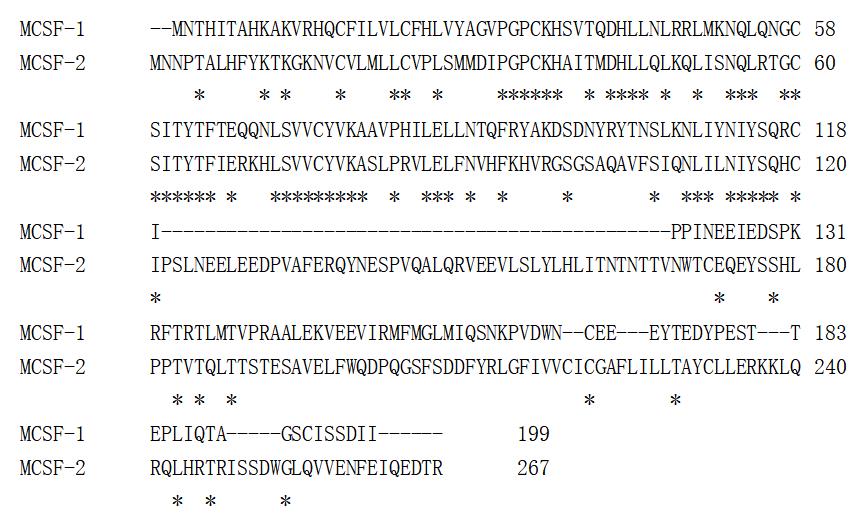


**Supplementary Figure 3.** Comparative analysis of goldfish *MCSF-1* and *MCSF-2* amino acid sequences using Clustal multiple sequence alignment. The asterisk (*) indicates the same amino acid between two sequences.


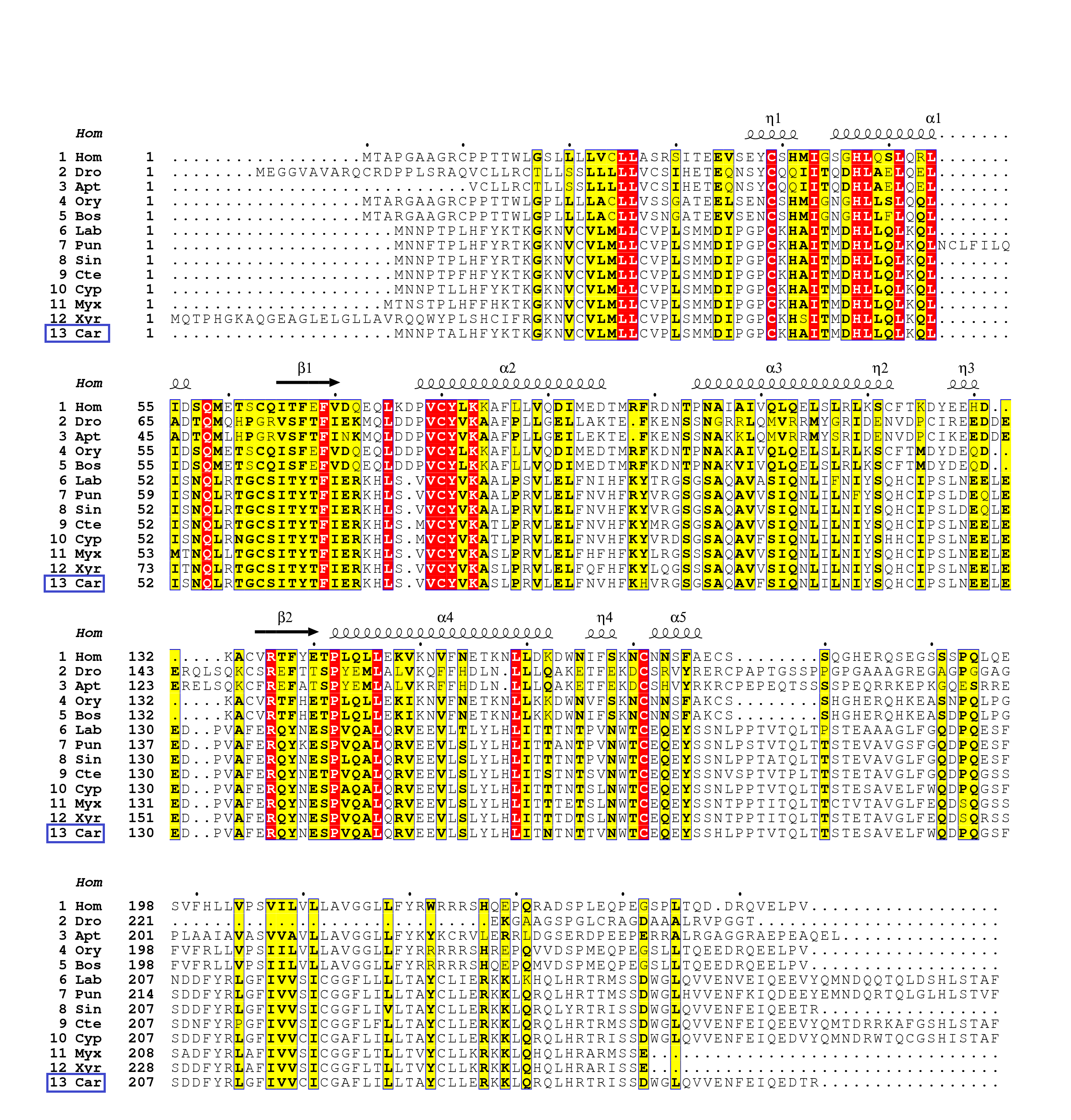


**Supplementary Figure 4.**  Multiple amino acid sequence alignment of gfMCSF-2 with other species *MCSFs*. The alignment was performed by CLUSTAL-W and images were generated using ESPript 3.0. The complete conserved amino acid residues are shown in red (100 % identical). The amino acid residues with a conservative degree higher than 75% are shown in yellow. The NCBI database accession numbers of these genes are listed in Table S2.


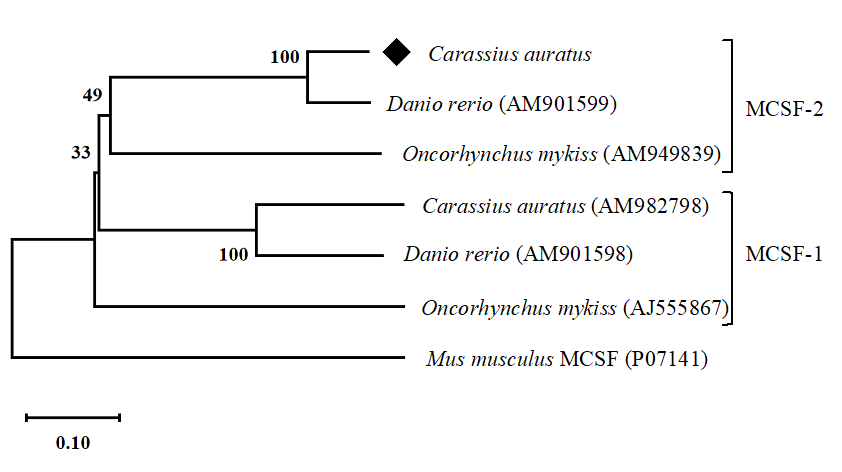


**Supplementary Figure 5.** Phylogenetic analysis of *MCSF-1* and *MCSF-2*. The phylogenetic tree was constructed using the neighbor-joining method with MEGA 11 software. Bootstrap

sampling was performed with 10,000 replicates.


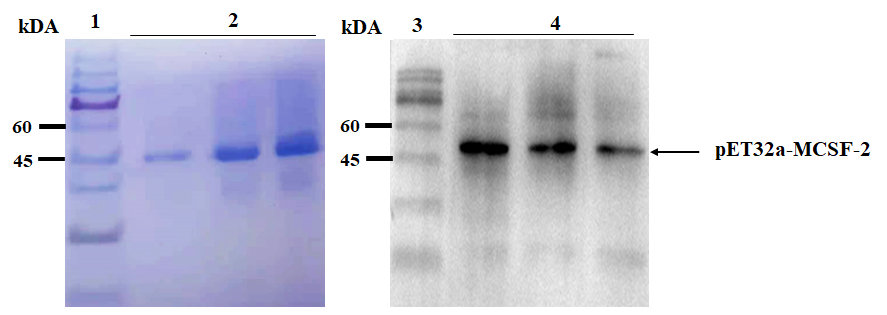


**Supplementary Figure 6.**  SDS-PAGE and western blot analysis of recombinant *MCSF-2*. Lanes 1 and 3: protein ladder; Lane 2: SDS-PAGE analysis of Coomassie blue stained of purified recombinant pET32a-MCSF-2; Lane 4: Western blot analysis of the purified recombinant pET32a-MCSF-2 using anti-His antibody.
